# Supplementary material for: An Internal-Electrostatic-Field-Boosted Self-Powered Ultraviolet Photodetector
Source: Nanomaterials (Basel). 2022 Sep 15;12(18):3200. doi: 10.3390/nano12183200 (PMC9503600; doi:10.3390/nano12183200)
Supplement: Supplementary file 1 [file nanomaterials-12-03200-s001.zip › nanomaterials-1895491-Supplementary.pdf]

Supporting Information

An electrostatic-potential-boosted self-powered ultraviolet photodetector

Dingcheng Yuan[a], Lingyu Wan[a*], Haiming Zhang[a], Jiang Jiang[a], Boxun Liu[a], Yongsheng Li[a], Zihan Su[a], Junyi Zhai[b*]

[a] Center on Nanoenergy Research, School of Physical Science and Technology, Guangxi University, Nanning, Guangxi 530004, P.R. China
[b] CAS Center for Excellence in Nanoscience, Beijing Key Laboratory of Micro-nano Energy and Sensor, Beijing Institute of Nanoenergy and Nanosystems, Chinese Academy of Sciences, Beijing, 100083, China

*Corresponding Author: lyw2017@gxu.edu.cn (LY. Wan), jyzhai@binn.cas.cn (JY. ZHai)

This file includes:

Supplementary Notes S1 to S4
Supplementary Figures S1 to S15
Supplementary Table S1

Supplementary Note S1

Quantitative analyse of the intermolecular transferred charges on TiO_2/PTFE and TiO_2/PDMS interfaces

Based on the first calculation principle and molecular dynamics simulation, the transferred charges between TiO_2, PTFE and PDMS molecules were quantitatively analysed and the electrostatic properties on the TiO_2/PTFE and TiO_2/PDMS interfaces were investigated. In this study, molecular structures were built by using software GaussianView 6.0 and all calculations were performed by using software Gaussian 09D011. Firstly, molecular formulas of TiO_2, PTFE and PDMS with a length of about 12 × 4 Å were constructed through GaussianView 6.0. After structure optimization by parametric method 6（PM6）in semiempirical theory, stable and reasonable molecular structures were obtained. Then, for a given distance between TiO_2, PTFE and PDMS molecules, the wave function of simulation model was analysed. Finally, the charge properties of each atom were calculated by utilizing the atomic dipole moment corrected Hirshfeld population (ADCH) charge analysis method and the charge transfer properties of TiO_2/PTFE and TiO_2/PDMS interfaces could be explored.

Figures S1 and S2 show the transferred charge properties in TiO_2/PTFE and TiO_2/PDMS interfaces at different interatomic distances. Figure S1a and Figure S2a display the diagrams of charge distribution of TiO_2 and PTFE and of TiO_2 and PDMS molecules at a distance of 2 Å. The statistical charged properties show that PTFE and PDMS molecules gain electrons and TiO_2 molecules lose electrons. At 2 Å, the PTFE with a molecule length of about 12 × 4 Å gain electrons of 0.5095 e ($e=-1.6\times10^{-19}$ C) and the PDMS molecules gain 0.3015 e. The simulation results reveal that PTFE and PDMS are negatively charged, while TiO_2 is positively charged. The PTFE gains more charges than that of PDMS. The varying charged characteristics with the interatomic distances between TiO_2 and PTFE, and TiO_2 and PDMS are displayed in Figure S1b and S2b. The smaller the intermolecular distance becomes, the stronger the chargeability is. The charge transfer mainly occurs at the interatomic distance range of 2-4 Å, as shown in Figures S1c and S2c.

Supplementary Note S2

Investigation of charge transfer properties in TiO_2/PTFE and TiO_2/PDMS interfaces through triboelectric nanogenerators (TENGs).

Based on the coupling of contact electrification (CE) and electrostatic induction, a triboelectric nanogenerator (TENG) composed of TiO_2 and PTFE was fabricated and the charge transfer properties between TiO_2 and PTFE surfaces were investigated. The experimental results revealed that the surface of PTFE is negatively charged and the surface of TiO_2 is positively charged after contacting. As shown in Figure S3a, the TENG consists of a TiO_2 film on glass substrate with ITO electrode and a PTFE film with Cu electrode. The TiO_2 film is prepared by using the same method as the photodetector and the PTFE film is bought from Japanese Teflon tape in Shenzhen, ChuKoh ASF-110FRtype. Two films with the size of 7×7 cm^2 contact and separate periodically by using a linear motor and the outputs of TENG provide the CE information between TiO_2 and PTFE surfaces. The working mechanism of TENG [2,3] is depicted in Figures S3a-e. At the original state (Figure S3a), there is no charge transfer happened before the contact of two films. When the PTFE and TiO_2 films contact each other (Figure 3b), electrons inject from TiO_2 into the PTFE, leaving positive charges on the TiO_2 surface. As the contact surfaces are separated, and an electric potential difference is then established between the two flims due to electrostatic induction. Because the potential of the TiO_2 is higher than that of the PTFE as shown in Figure S3c, electrons will be transferred from the Cu electrode to the ITO electrode, thereby generating a current from ITO to Cu. An open-circuit voltage V_{oc} will keep increasing and reach the maximum value until an equilibrium will be reached as shown in Figure S3d. Once the TENG is being close again, the electric potential difference starts to decrease as the two films get closer to each other Figure S3e. The V_{oc} will drop from the maximum value to zero when a full contact is made again between the flims. A reduction of the gap distance would make the PTFE a higher electric potential than the TiO_2. Consequently, the electrons will flow from ITO electrode to Cu electrode, producing a reverse current until achieving a new equilibrium (Figure S3b).

Connecting the ITO and Cu electrodes with the positive and negative electrodes of the Keithlay 6514 electrometer, above output characteristics of TENG were observed. After the

initial contact, Figures S4 a-c shows the transferred charges, output currents and voltages of TENG in the releasing and pressing stages. Positive charges of 110 nC are transferred from ITO to Cu electrode in the releasing process and the output current and voltage are ~ 4 μA and 195 V, respectively. In the pressing stage, opposite current is observed to be about 5 μA. The experimental results indicated when TiO_2 and PTFE are in contact, the TiO_2 surface is positive charged whlie the PTFE surface is nagetive charged and an electrostatic field can be yeilded. That is agree with the simulation results in the Note. S1.

During the preparation of our device, PTFE is deposited on the TiO_2 surface by magnetron sputtering, and the growth process corresponds to the pressing stage of TENG. The charge transfer occurs between PTFE and TiO_2 molecules. Since PTFE is an electret material, it traps and stores electrons and a built-in electrostatic field is gradually formed with the continuous deposition of PTFE on the surface of TiO_2. As for the holes in TiO_2, they can diffuse towards the ITO due to the concentration difference.

Similar properties were observed for the TENG composed of TiO_2 and PDMS, as shown in Figures S4d-f. But its output is ~ 10 times lower than that of TENG of TiO_2 and PTFE. Correspondingly, the detector with PTFE exhibits better photodetection performance than the detector with PDMS.

References

1. Wang, Z. L.; Wang, A. C., "On the origin of contact-electrification." Materials Today 30, 34-51 (2019).

2. Lin, Z.-H.; Cheng, G.; Yang, Y.; Zhou, Y. S.; Lee, S.; Wang, Z. L., "Triboelectric Nanogenerator as an Active UV Photodetector." Advanced Functional Materials 24 (19), 2810-2816 (2014).

3. Wang, Z. L.; Lin, L.; Chen, J.; Liu, S.M.; Zi, Y.L., Triboelectric Nanogenerator, first ed., Science Press, Beijing, 2017.

Supplementary Note S3

The measurements of internal resistances for photodetectors through impedance matching method.

The high resistivity of the PTFE layer made a major contribution for reducing the dark current of the device. We did tests to evaluate the internal resistances of the devices with and without PTFE. Taking the photodetector in the light-on state as a power supply, we use a rheostat as an external load, and estimate the internal resistance of the device through the method of impedance matching. The equivalent electric circuit diagrams of testing are shown in Figures S5a. While the devices with PTFE and without PTFE are illuminated by a UV light with the wavelength of 365 nm and the optical power density of 15.94 mW/cm^2, the currents and output powers of loads with different resistances are shown in Figures S5b-c. At maximum power output, the load resistance is equal to the internal resistance of the power supply. It can be seen that the device with PTFE has the highest power at a load resistance of ~ 200,000 Ω, while the device without PTFE has peak power at a resistance of ~ 15 Ω. The PTFE layer increased the internal resistance of device by ~ 1.3×10^5 times, which basically matches the order magnitude of dark current reduction ($3.60\times10^{-6}/8.03\times10^{-12}\approx4.5\times10^5$).

Supplementary Note S4

Calculation of photovoltage increment for the device with PTFE.

The improving factor (k_v) of photovoltage is defined as the ratio of voltage increments. It can be calculated by the expression as follows:

$$\mathrm{k}_v = \frac{V_{p1}-V_{d1}}{V_{p0}-V_{d0}} = \frac{0.02-8.52\times10^{-5}}{4.51\times10^{-5}-1.52\times10^{-5}} = 665,$$

where, V_{p1} and V_{d1} are the photovoltage and the dark voltage for the device with PTFE, and V_{p0} and V_{d0} are the photovoltage and the dark voltage for the device without PTFE, respectively.

Supplementary Figures

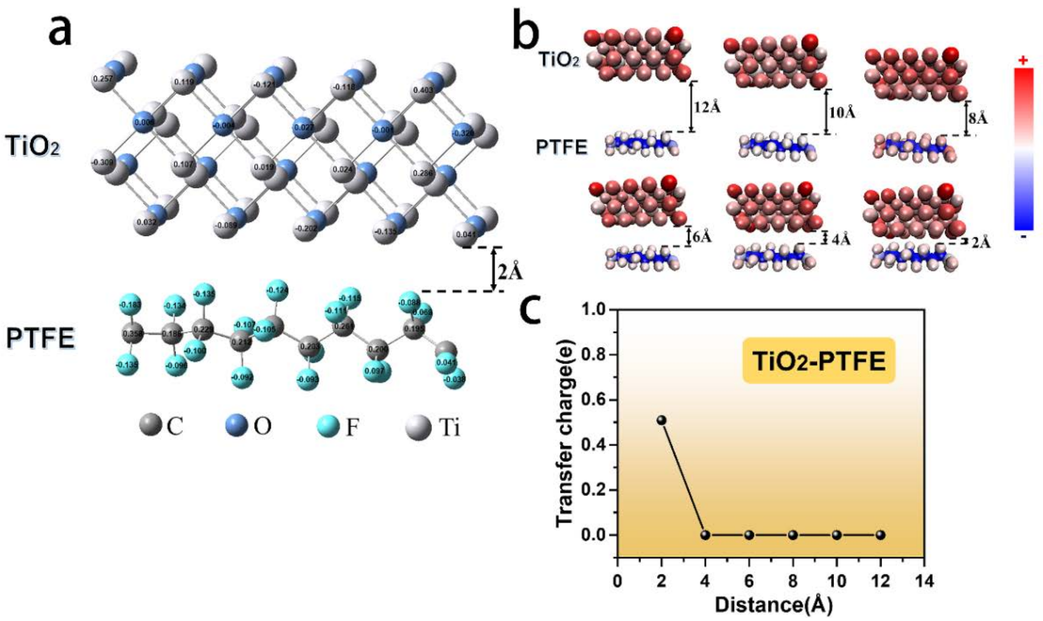

Figure S1. Results of molecular dynamics simulation and charge properties of TiO_2 and PTFE molecules. (a) The diagram of charge distribution of TiO_2 and PTFE molecules as the interatomic distance is 2 Å. (b) The diagram of electrification properties at different interatomic distances (from 2 Å to 12 Å). (c) The relationship between the transferred charges and interatomic distances.

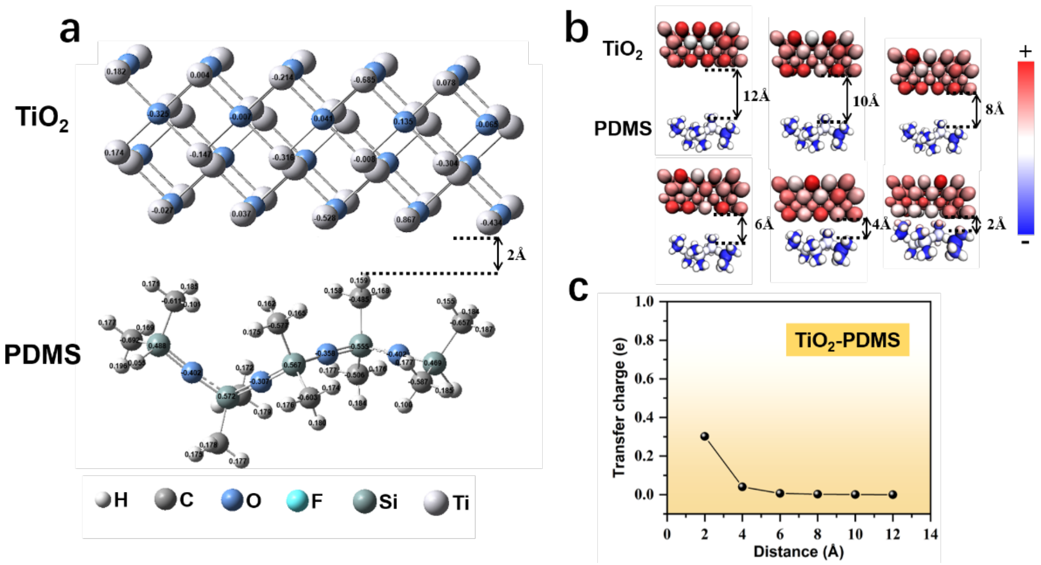

Figure S2. Results of molecular dynamics simulation and charge properties of TiO_2 and PDMS molecules. (a) The diagram of charge distribution of TiO_2 and PDMS molecules as the interatomic distance is 2 Å. (b) The diagram of electrification properties at different interatomic distances (from 2 Å to 12 Å). (c) The relationship between the transferred charges and interatomic distances.

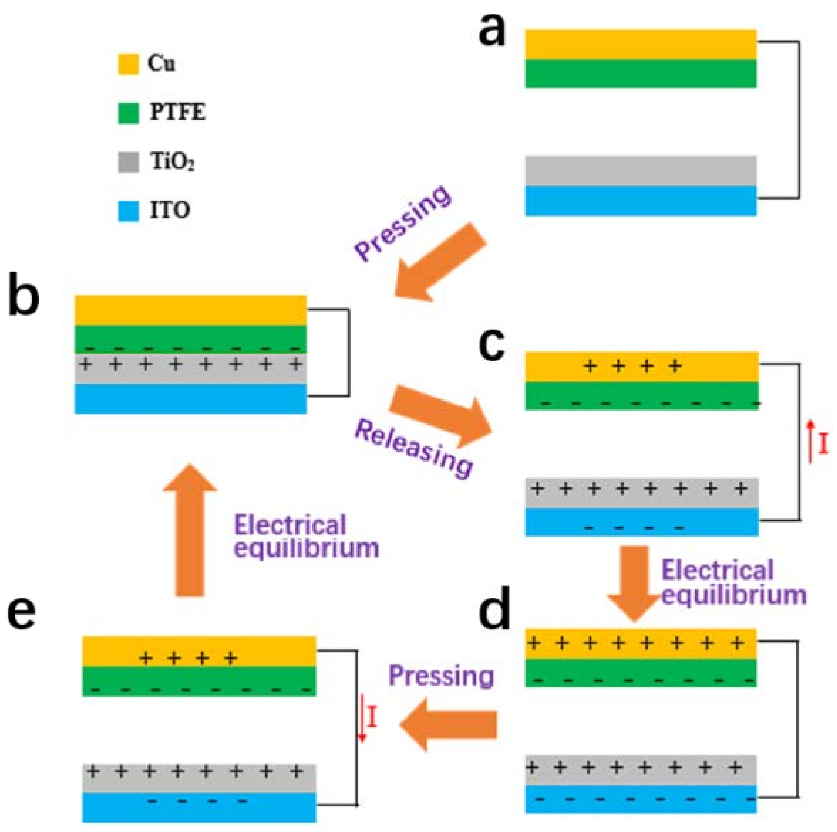

Figure S3. Working mechanism of TENG composed of PTFE and TiO_2 films.

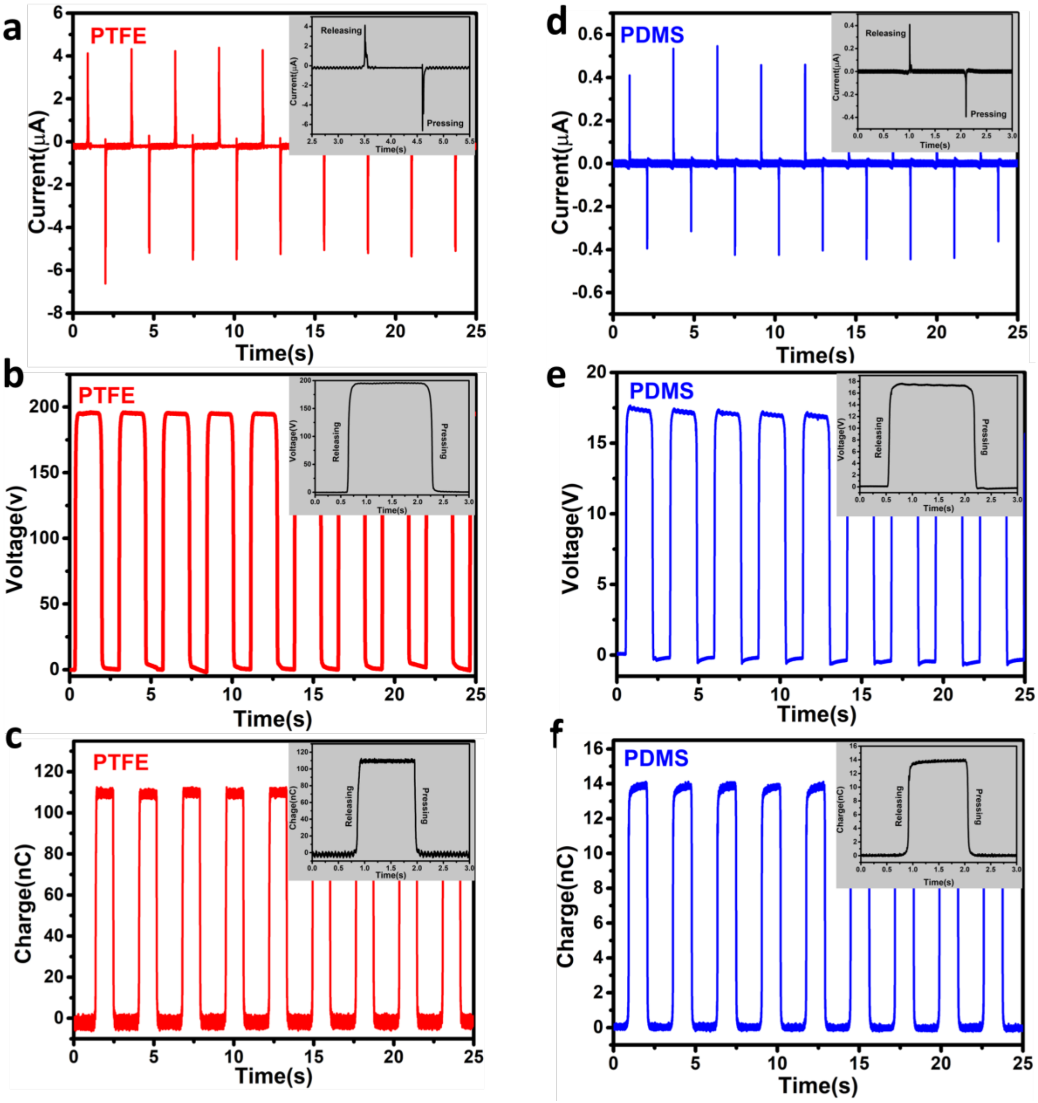

Figure S4. (a) Output voltage, (b) output current, and (c) transferred charge of the TENG composed of TiO_2 and PTFE. (d) Output voltage, (e) output current, and (f) transferred charge of the TENG composed of TiO_2 and PDMS.

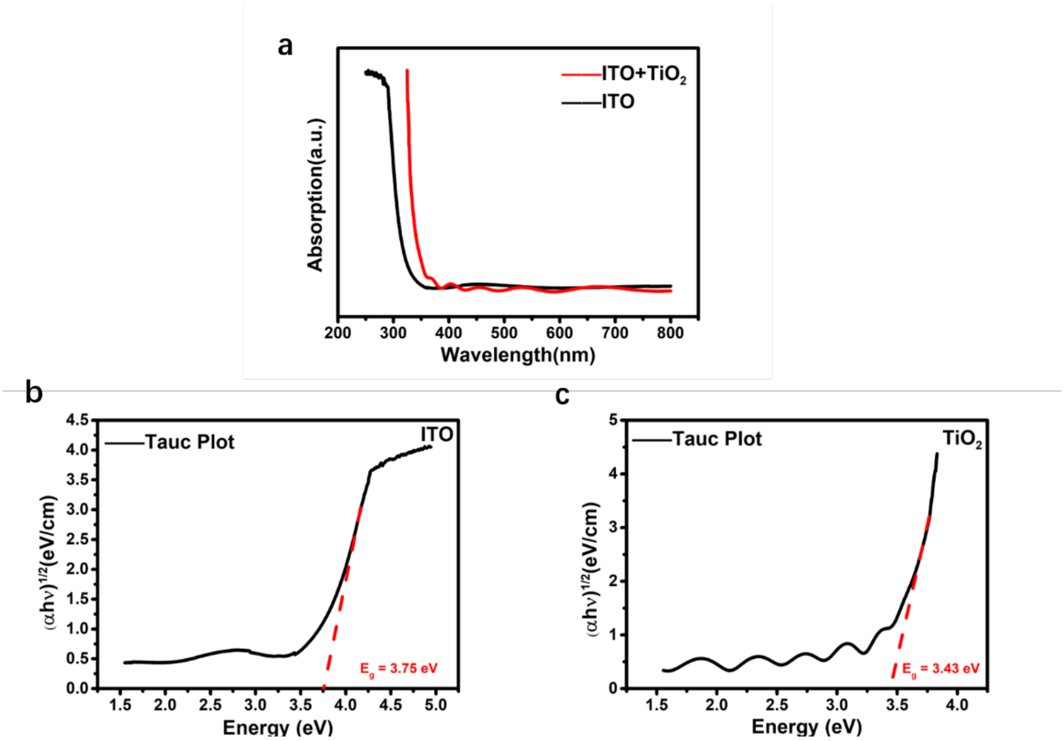

Figure S5. (a) Absorption results of the ITO with glass substrate and the sample after the growth of TiO_2 film. (b) Band gap values of ITO from Tauc's plots. (c) Band gap values of TiO_2 from Tauc's plots.

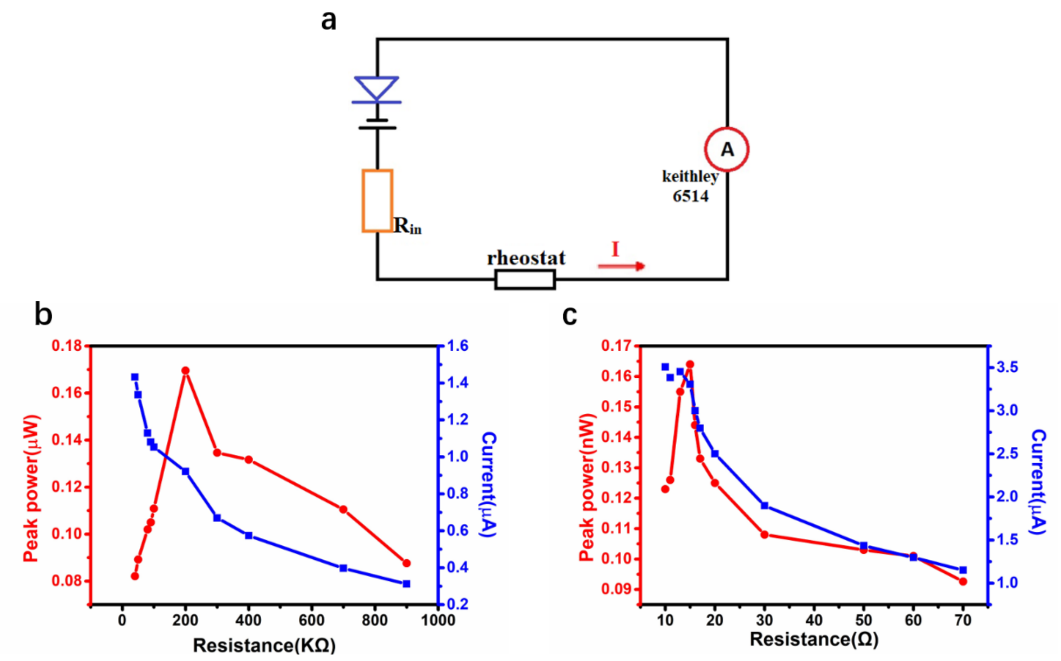

Figure S6. (a) Equivalent circuit diagram of internal resistance testing for the device. (b) Out power of the the device with PTFE under various loads. (c) Output power of the device without PTFE under various loads.

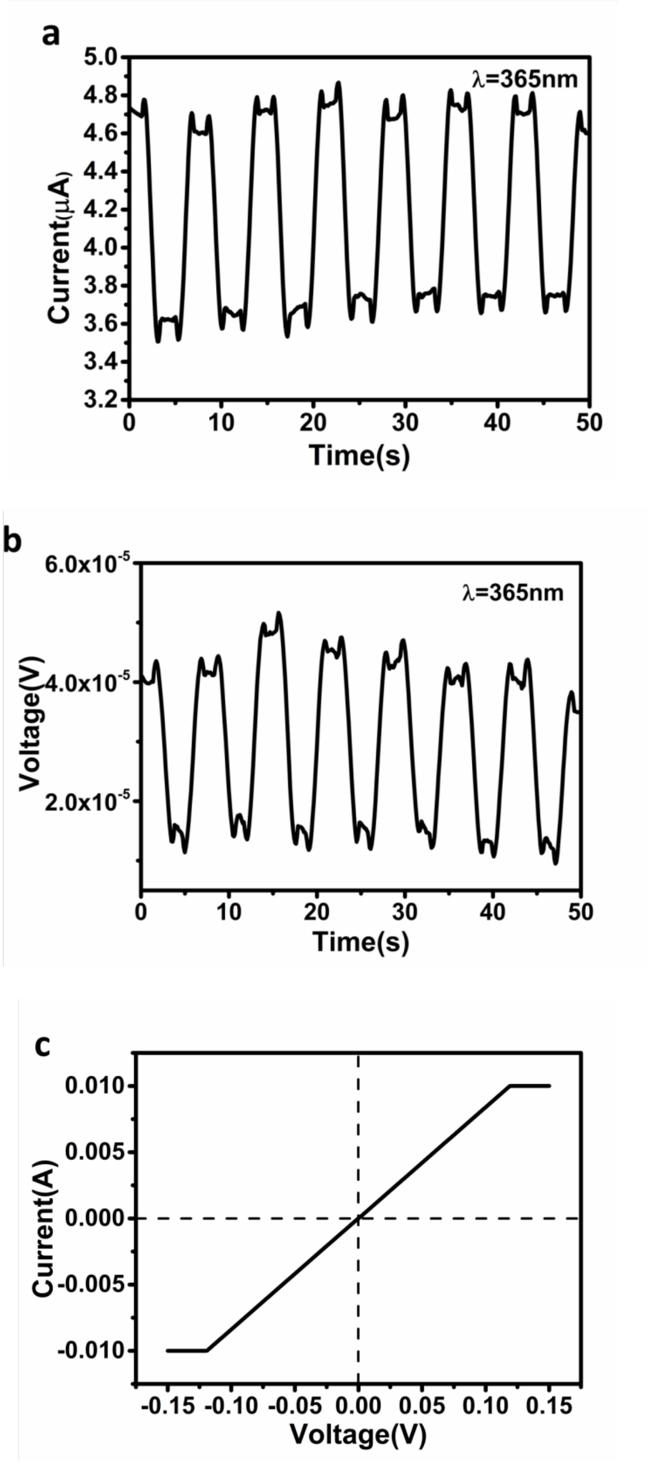

Figure S7. Photodetection performances of conventional ITO/TiO_2/Cu junction device without PTFE. Photocurrent (a) and photovoltage (b) of device without PTFE; (c) I-V curve connecting the TiO_2 and the Cu electrode for the device without PTFE.

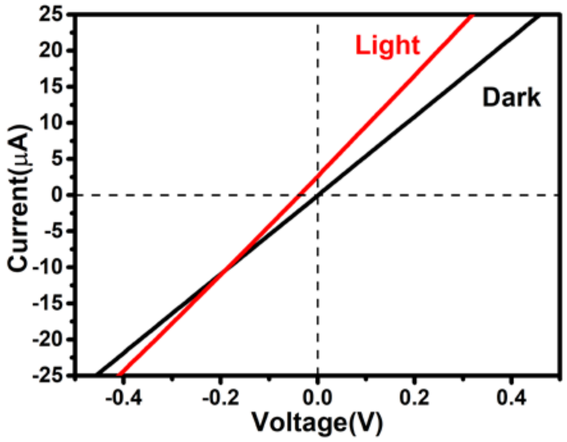

Figure S8. Current-voltage (I-V) curves for the ESPB-SP photodetector under 365 nm light illumination with intensity of 15.94 mW/cm^2 and dark condition.

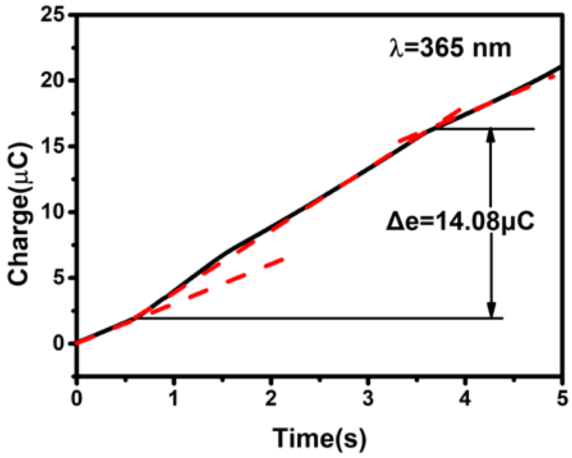

Figure S9. The amount of transferred charges in each illumination for the photodetector without PTFE.

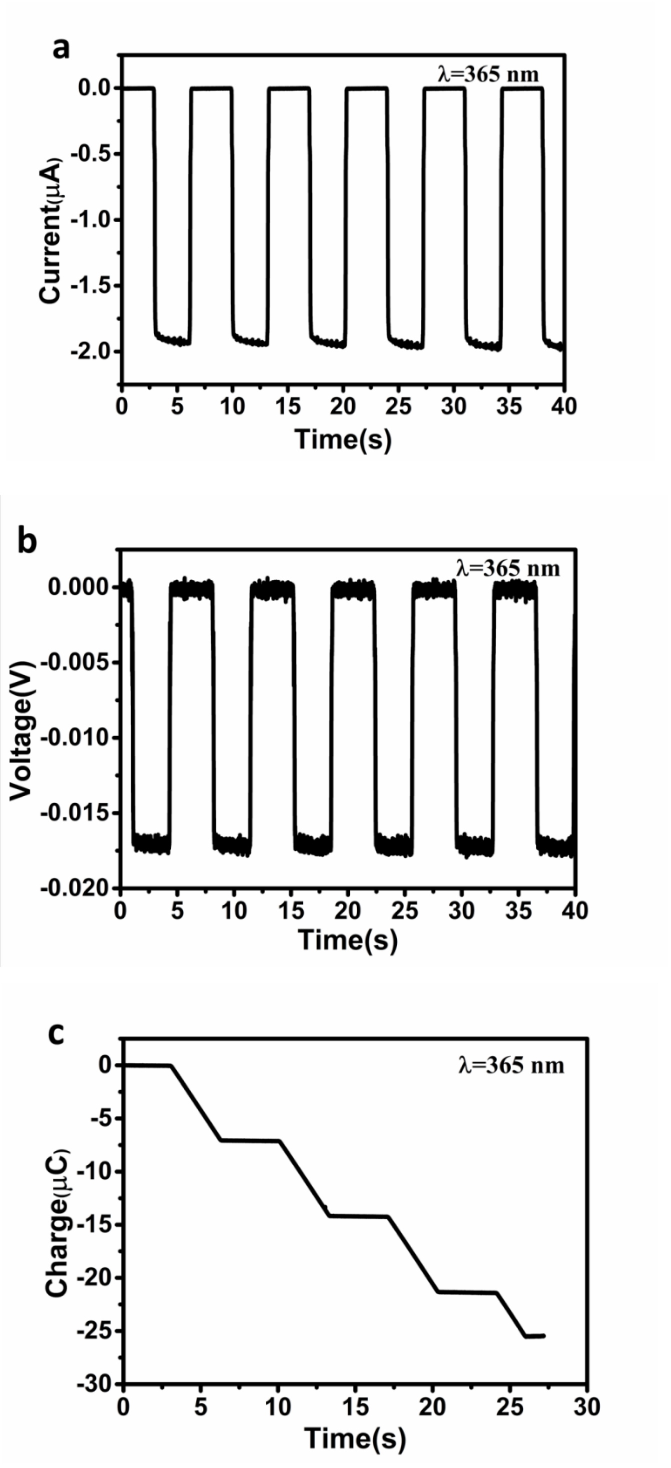

Figure S10. Performance of ESPB-SP device with reversed the positive and negative connections between ITO and Cu electrodes. (a) Output currents; (b) Output voltages; (c) The transferred charges under the illumination of 365-nm light-emitting diode (LED)

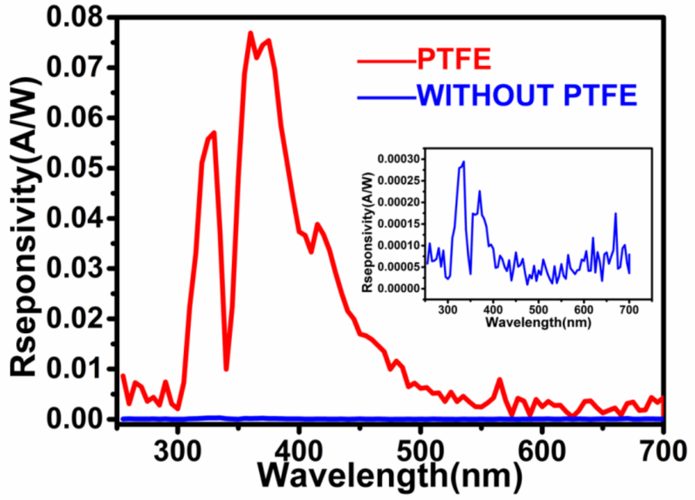

Figure S11. Comparison of the responsivity of devices with and without a PTFE layer

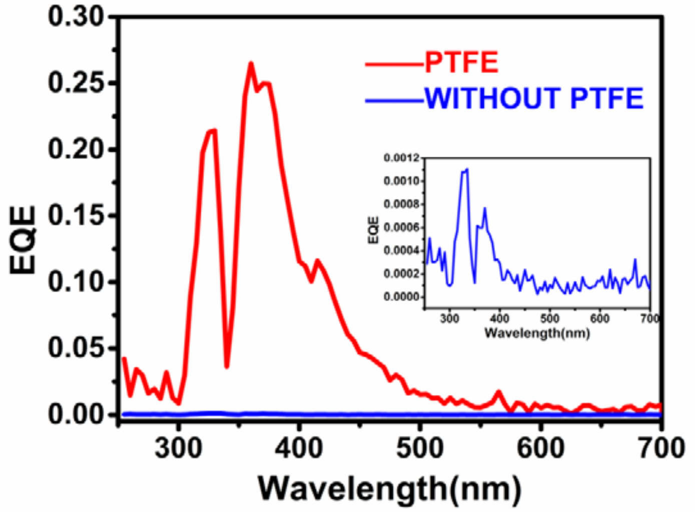

Figure S12. Comparison of the EQE of devices with and without a PTFE layer

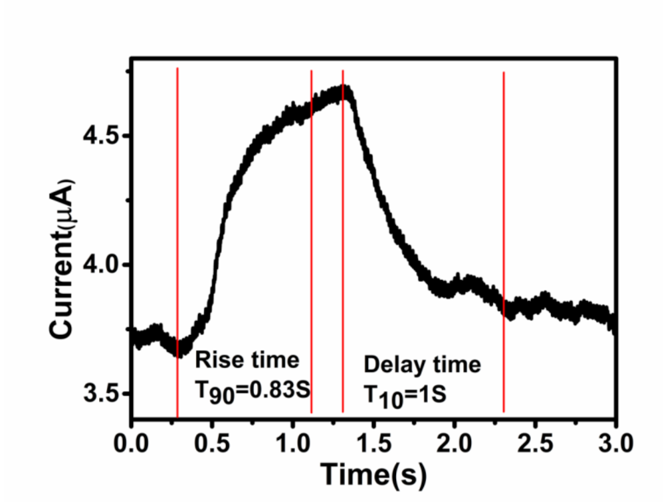

Figure S13. Response speed of conventional device without a PTFE layer.

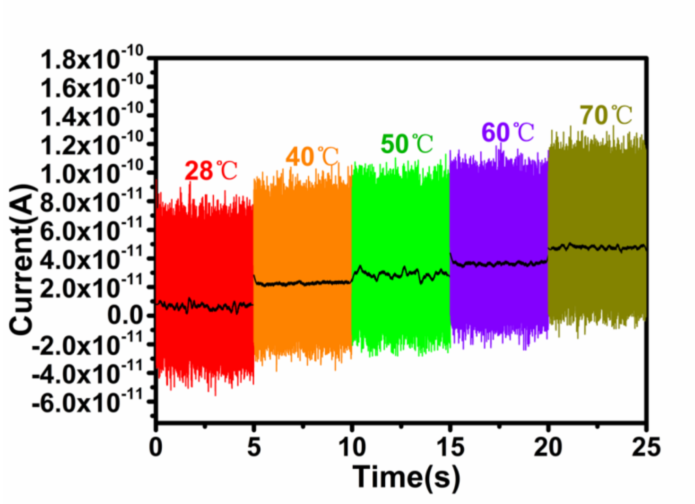

Figure S14. Dark current of the ESPB-SP device from room temperature to 70°C

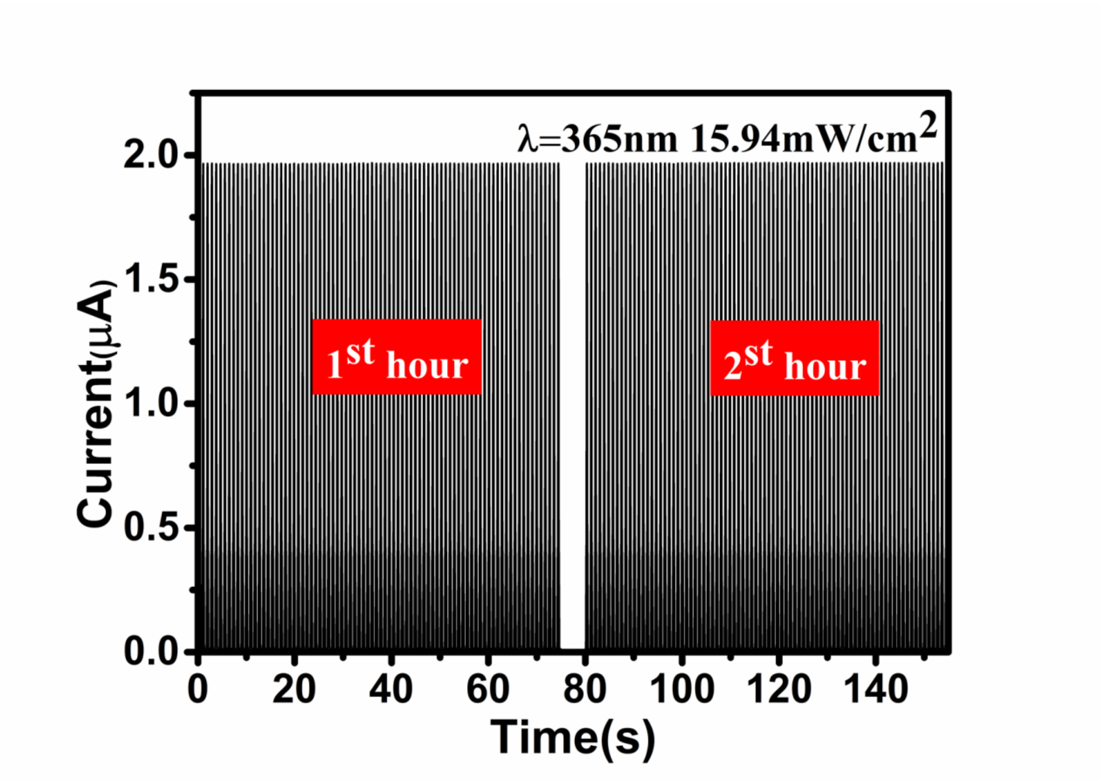

Figure S15. Repeatability measurement for the ESPB-SP device with PTFE for 2 h.

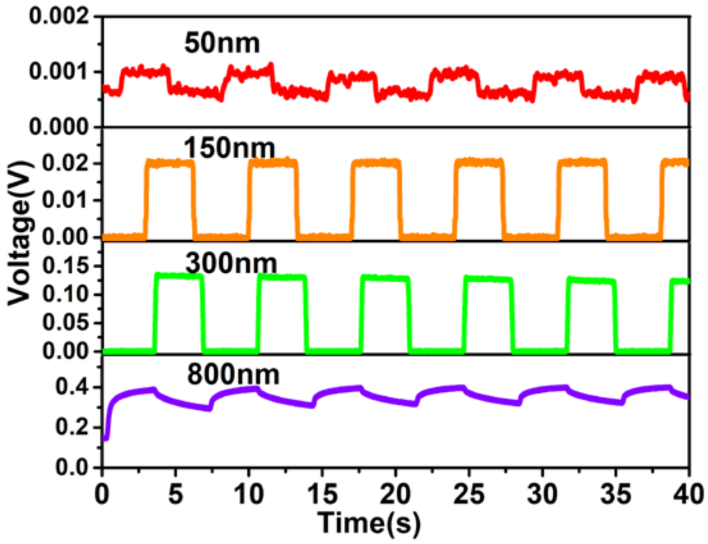

Figure S16. Photovoltage of the device with different PTFE thicknesses (50, 150, 300, and 800 nm).

Supplementary Table S1

Table S1. Performance comparison of three PDs (irradiated by a UV light with the wavelength of 365 nm and the optical power density of 15.94 mW/cm^2). I_p/I_d : the ratio of photocurrent to dark current; V_p/V_d: the ratio of photovoltage to dark voltage.

Devices	Dielectric materials	Internal resistance (Ω)	Photo-current (A)	Dark current (A)	I_p/I_d	Photovolt-age (V)	Dark voltage (V)	V_p/V_d
ITO/TiO_2	–	~ 15	4.76×10^{-6}	3.67×10^{-6}	1.30	4.51×10^{-5}	1.52×10^{-5}	2.97
ESPB-SP	PTFE	~ 200,000	2.00×10^{-6}	8.03×10^{-12}	2.49×10^{5}	2.00×10^{-2}	8.52×10^{-5}	235
	PDMS	~ 800	5.02×10^{-7}	8.36×10^{-8}	6.00	2.03×10^{-4}	1.22×10^{-4}	1.67
